# A porcine commotio retinae model for preclinical evaluation of posttraumatic photoreceptor degeneration

**DOI:** 10.1172/jci.insight.192799

**Published:** 2026-01-13

**Authors:** Juan Amaral, Irina Bunea, Arvydas Maminishkis, Maria M. Campos, Francesca Barone, Rohan Gupta, Mitra Farnoodian, Jonathan Newport, M. Joseph Phillips, Ruchi Sharma, David M. Gamm, Kapil Bharti, Richard J. Blanch

**Affiliations:** 1 National Eye Institute (NEI)/Translational Research Core/Ophthalmic Genetics and Visual Function Branch (OGVFB), Bethesda, Maryland, USA.; 2 NEI/Ocular and Stem Cell Translational Research Section/OGVFB, Bethesda, Maryland, USA.; 3 NEI/Biological Imaging Core/Histology Core, Bethesda, Maryland, USA.; 4 NEI/Laboratory of Sensorimotor Research, Bethesda, Maryland, USA.; 5 Waisman Center,; 6 McPherson Eye Research Institute, and; 7 Department of Ophthalmology, University of Wisconsin–Madison, Madison, Wisconsin, USA.; 8 Academic Department of Military Surgery and Trauma/Royal Centre for Defense Medicine, Birmingham, United Kingdom.; 9 Neuroscience and Ophthalmology, School of Infection, Inflammation, and Immunology, University of Birmingham, Birmingham, United Kingdom.; 10 Department of Ophthalmology, University Hospitals Birmingham NHS Foundation Trust, Birmingham, United Kingdom.

**Keywords:** Neuroscience, Ophthalmology, Neurodegeneration, Stem cell transplantation

## Abstract

Commotio retinae (CR) resulting from retinal trauma can lead to focal photoreceptor degeneration and permanent vision loss. Currently no therapies exist for CR-induced retinal degeneration, in part because of the lack of a large-animal model that replicates human injury pathology and allows testing of therapeutics. Severe CR is clinically characterized by subretinal fluid and focal photoreceptor outer nuclear layer thinning. To develop a porcine CR model, we developed a laser-guided projectile apparatus and optimized projectile delivery procedure using porcine cadaveric eyes embedded in a 3D-printed porcine skull. Scleral and corneal impacts resulted in retinal damage consistent with patient injury, but corneal impacts also led to cornea damage and opacification, which precluded follow-up imaging. In live porcine eyes, scleral impacts of 39.5 m/s induced transient blood-retinal barrier breakdown evidenced by subretinal fluid on optical coherence tomography (OCT), leakage observed on fluorescein and indocyanine green angiography, and transient photoreceptor outer segment disruption seen by OCT and multifocal electroretinography. Impacts above 39.5 m/s induced longer-lasting photoreceptor degeneration but only transient blood-retinal barrier breakdown. This porcine model, combined with clinically relevant imaging and diagnostic modalities, will be valuable for testing the safety and efficacy of therapies to restore vision after focal photoreceptor degeneration.

## Abstract 2

Swine model of post traumatic outer retinal degeneration using clinical imaging modalities to evaluate its evolution. Model will be valuable for testing cell-based therapies.

## Introduction

Traumatic retinopathy is a significant cause of vision loss and blindness, with eye injury–associated vision impairment affecting 4.5 per 1,000 Americans. Among those affected, 5.1 per 1,000 experience unilateral blindness, and 4.5 per 10,000 have bilateral blindness (1–3). Globally, 55 million eye injuries occur annually, resulting in bilateral visual impairment in 2.3 million people and unilateral blindness or low vision in nearly 19 million individuals (4, 5). Furthermore, traumatic vision loss is often acute (4), affects relatively younger individuals, is associated with occupational and psychiatric complications, and contributes significantly to lost productivity and reduced quality of life (6).

One form of traumatic retinopathy is commotio retinae (CR), a condition affecting the outer retina, associated with temporary or permanent visual function loss following a closed-globe injury (7–11). The incidence of CR in the civilian population is approximately 0.4%, but it accounts for up to 15% of military ocular trauma cases and leaves a significant number of veterans with lifelong visual impairment (8, 10, 12). In real-world scenarios, the location of impact leading to CR is sporadic, unpredictable, and often unknown. Depending on the location, the damage can also be variable. Of concern are the CR cases in which the macula is involved; in such cases visual acuity can be significantly reduced without any treatment possibility. Macular CR can occur after anterior segment trauma (contrecoup) or direct scleral impact (13, 14). After macular CR, 15%–20% of patients suffer permanent visual impairment, primarily related to photoreceptor degeneration (7, 8, 15). Rats, rabbits, cats, pigs, and rhesus and owl monkeys have been used as animal models for CR (15–27); however, none fully or consistently replicate the macular pathology observed in human injury. Some models, such as rabbits and rats, lack a macula homolog, while others, such as pigs and nonhuman primates that have a macula homolog, were developed predominantly with direct peripheral injuries rather than central (where energy of impact is transmitted to the macular region) as seen in human CR pathology. Another unresolved aspect of CR injury is the nature of Berlin’s edema, which has been described as a hallmark feature of CR pathology (28). But there is ongoing debate about the degree to which the outer blood-retinal barrier is disrupted following CR injury (16, 18, 20, 23, 29, 30). The inability heretofore to perform clinically relevant longitudinal live imaging modalities has further limited translation of previous CR models to test human-relevant treatment modalities. Moreover, because CR injury can affect a relatively large retinal area — including the entire macula (up to 20 mm^2^) — there is a clear need for a large-animal model of CR that accurately replicates human closed-globe macular injury. Such a model would enable proper characterization of the injury response and facilitate testing of clinically relevant imaging techniques and allow preclinical evaluation of regenerative therapies aimed at replacing lost photoreceptors and/or restoring photoreceptor function through other approaches.

Pigs are an ideal preclinical animal to develop such a model, as the porcine eye lacks a tapetum, has a human-comparable average axial length of 23.9 mm, and has a holangiotic retinal blood supply with a capillary meshwork of similar caliber that supplies identical retinal layers to those in human eyes (31). While pigs do not have a macula, they have a central visual streak rich in cone photoreceptors (31, 32). Because of their comparability to human eyes, pig eyes are suitable for surgical procedures such as vitrectomy and subretinal transplantation of large constructs that can cover a significant portion of the macula (33, 34). Furthermore, pigs are significantly more cost-effective and easier to handle and obtain as compared with nonhuman primates (33, 35, 36).

Using a scleral impact approach, we developed a closed-globe macula injury–specific CR pig model to characterize the acute and chronic injury response. Scleral impact allowed us to test clinically relevant imaging modalities not feasible with corneal impact (36, 37), such as optical coherence tomography (OCT), OCT angiography (OCTA), fluorescein and indocyanine green angiography (FA/ICGA), and multifocal electroretinography (mfERG), to evaluate and diagnose the cone photoreceptor response to injury. Our data suggest that the porcine CR model closely mimics the human closed-globe macular CR injury. Our model showed retinal whitening, preretinal hemorrhage, transient outer blood-retinal barrier breakdown, and progressive photoreceptor outer segment degeneration — symptoms seen in patients with a CR injury. This clear longitudinal analysis is useful for the development of treatments for severe macular CR and may aid in the development of treatments for other forms of outer retinal degenerations.

## Results

### Development of a pressure application device for inducing closed-globe CR injury.

**Figure 1:**
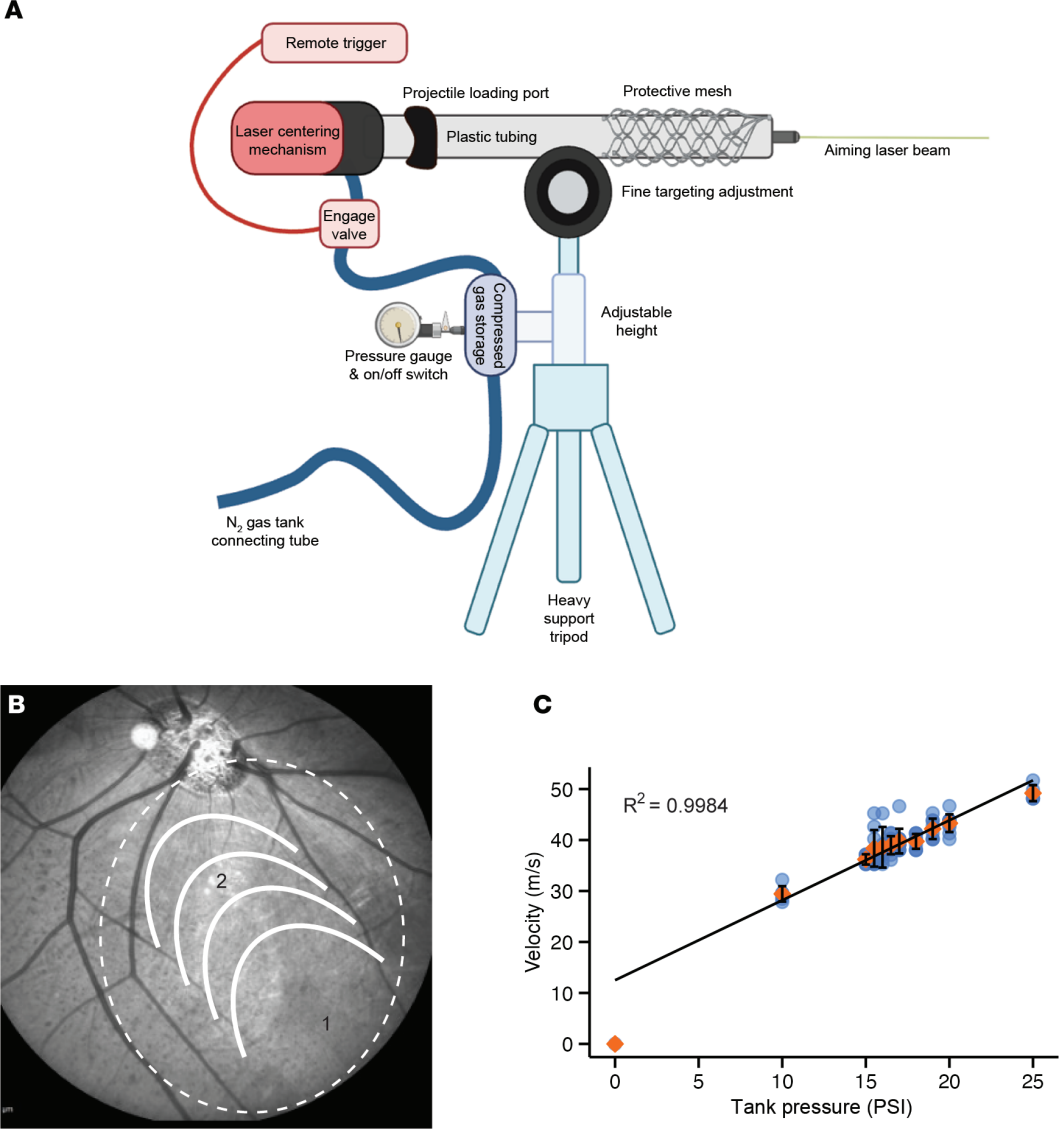
Development of a pressure application device for inducing closed-globe commotio retinae in porcine eyes. (**A**) The pressure application device (PAD) contains a plastic tube, a projectile loading port, and an aiming laser beam. The PAD connects to a nitrogen tank and a pressure manometer, allowing control of pressure (measured in psi) used to propel the projectile (~12 mm/0.75 g). A remote trigger releases the pressure to propel the projectile. (**B**) Porcine fundus infrared photograph showing the visual streak (white dashed circle). “1” indicates the site of projectile impact on peripheral retina. “2” (semicircles) indicates the projected path of the shockwave generated by the impact, leading to indirect visual streak damage. (**C**) Graph shows the average of projectile speed (m/s) measurements as a function of nitrogen gas pressures (psi) ranging from 10.0 to 25.0 psi (gauge pressure). Results were analyzed using 1-way ANOVA. The standard deviation of the mean was used to estimate uncertainty in projectile speed for each tank pressure.

To develop a reproducible closed-globe CR porcine model that mimics human macular CR injury, we developed a pressure application device (PAD) that injures the eye using a fixed-diameter projectile impact. To induce injury, PAD delivers predetermined energy by propelling a plastic projectile (~12 mm) toward the pig eye at a measured speed using a laser-guided mechanism. The PAD was designed as a closed pneumatic system that operates using compressed nitrogen gas to generate precise pressure, measured in pounds per square inch (psi), that can be reproducibly applied to a propel a plastic ball at a specific speed, measured in m/s. To accurately target the injury location at the visual streak, the eye fundus was visualized using binocular indirect ophthalmoscopy, and different projectile impact areas (cornea, limbus, and sclera) were tested. The plastic ball is “loaded” into an acceleration tube using the loading port, then propelled using pressure from the compressed nitrogen gas and a solenoid-actuated fast-opening valve with remote trigger; the tube is aimed at the desired location using the laser beam (Figure 1A, Supplemental Video 1, and Supplemental Figure 1, A and B; supplemental material available online with this article; https://doi.org/10.1172/jci.insight.192799DS1). The impact of the plastic ball transfers its kinetic energy to the eye, modeling blunt-force or concussive injury such as after a blast (Figure 1B). To determine projectile speed reproducibility, we evaluated linearity between psi and projectile speed (Supplemental Figure 1B) and confirmed a linear relationship between psi and the projectile speed within the range of pressures tested for the projectile release (Figure 1C). These data confirm our ability to deliver the projectile consistently at a specific speed.

### Ex vivo evaluation of PAD-induced CR.

**Figure 2:**
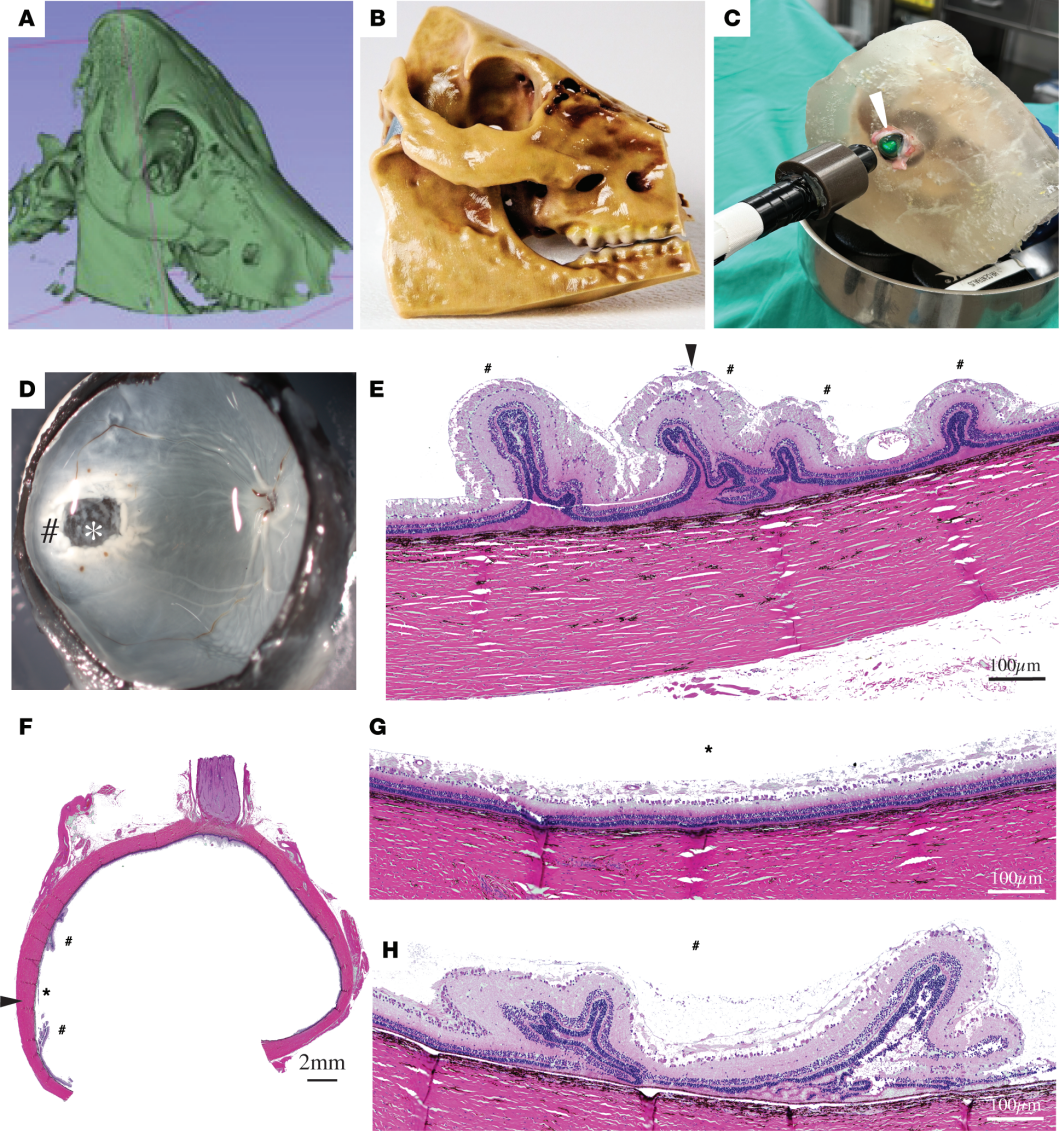
Ex vivo evaluation of CR injury using 3D-printed porcine skull. (**A**) CT scan–assisted 3D rendering of a pig skull. (**B**) 3D-printed model of a pig skull from 3D rendering generated in **A**. (**C**) 3D-printed pig skull with a fitted cadaveric pig eye in the orbit along with non-gelatin-based 10% ballistic gel. The PAD laser (green) is aimed at the cadaveric pig eye (arrowhead). (**D**) Gross specimen view after the CR injury showing the impact area in the sclera (*) and a surrounding whitened area (#). (**E**) Hematoxylin and eosin–stained (H&E-stained) sections from retinal region corresponding to the corneal impact (#) showing retinal folds in the visual streak. Arrowhead shows the impact direction in the cornea. (**F**–**H**) H&E-stained eye section showing the scleral impact zone (arrowhead, **F**), impacted retina (*) in lower (**F**) and higher (**G**) magnifications, and retinal region with folds (#) surrounding the impact area, in lower (**F**) and higher (**H**) magnifications. Scale bars: 100 μm in **E**, **G**, and **H**; 2 mm in **F**. *N* = 3 eyes per condition.

To develop an animal model with reproducible macular CR injury, we set out to optimize the injury location that would consistently induce damage to the ellipsoid zone (inner/ outer segments of the photoreceptors) and photoreceptor outer nuclear layer (ONL) in the pig’s visual streak. With the goal of reducing the number of animals, we chose to perform initial testing of optimal injury location and projectile speed ex vivo on cadaveric pig eyes. Since slaughterhouse eyes lack the orbital support (muscle, fat, ligaments, optic nerve) to reduce artificiality of an ex vivo model and to mimic as closely possible the in vivo environment, we developed a 3D-printed pig skull in which we mounted the cadaveric pig eyes. A high-resolution computed tomography (CT) scan of a pig head was performed and was used to construct a 3D image of the pig skull using the CT scan software (Figure 2A). This 3D image was used to 3D-print a pig skull. The skull was printed commercially using material that has similar mechanical properties to bone (https://www.anatomicalworldwide.com) (Figure 2B). A ballistic gel (see Methods) made from synthetic gelatin was used to fill the 3D-printed pig skull, mimicking the elastic properties of the orbital and cranial tissues, thus explicitly helping to assess the impact of projectile kinetic energy transfer to the eye simulating what happens in a live animal and human injury. Freshly obtained cadaveric pig eyes were placed in the eye sockets of the 3D-printed pig skull (Figure 2C and Supplemental Figure 2, A and B). Despite lacking anatomical structures around the eye (muscles, blood vessels, and the fat deposits), these cadaveric eyes provided the first best approximation of injury location and impact intensity, allowing us to rule out conditions incompatible with our goal of central retinal injury with minimal corneal damage. PAD was used to deliver projectiles at different speeds to determine the optimal speed and the optimal impact location (Figure 2C). Injury damage was evaluated by gross evaluation in dissected eyes and by histology (Figure 2, D–H, and Supplemental Figures 2 and 3). We first tested the hypothesis that direct corneal impacts can lead to visual streak damage. With projectile speed of 40.5 m/s, although we detected damage to the retina and the visual streak, the impact caused retinal folds and discernible damage to the cornea with epithelial displacement (Figure 2, D and E, Supplemental Figure 2, C and D, and Supplemental Table 1). Since retinal folds were not a desired outcome and corneal damage would preclude longitudinal live imaging, we did not pursue this approach further. Next, we asked whether projectile impacts at the limbus would damage the retina. Unexpectedly, limbal impacts with projectile speed of 40.5 m/s induced only a peripheral damage to the cornea, and a peripheral retinal dialysis (retinal tear at the ora serrata) was also seen (Supplemental Figure 2, E and F, and Supplemental Table 1). This led us to test whether direct scleral impacts could result in desired retinal damage. With progressively increasing projectile speed from 33 to 40 m/s, damage to the retina at the impact site increased from displacement of retinal layers at 33 m/s to edema and fibrosis at 35.7 m/s to almost complete retinal atrophy at 40 m/s (Supplemental Figure 3, A–E). In comparison, in the visual streak, projectile speeds at and below 39.5 m/s caused desired damage to the ellipsoid zone and the ONL, whereas speed of 40 m/s or more caused atrophic changes and retinal folds, as well as random preservation of retinal structures outside the impact area (Figure 2, F–H; Supplemental Figure 3, F–J; and Supplemental Table 1). Based on our analysis of cadaveric pig eyes, we hypothesized that living pig eyes would be more sensitive to scleral damage, so relatively lower velocities would be required to damage the visual streak area and minimize collateral damage to the anterior segment of the eye that would preclude longitudinal post-injury evaluations.

### Acute outer blood-retinal barrier damage in PAD-induced CR.

**Figure 3:**
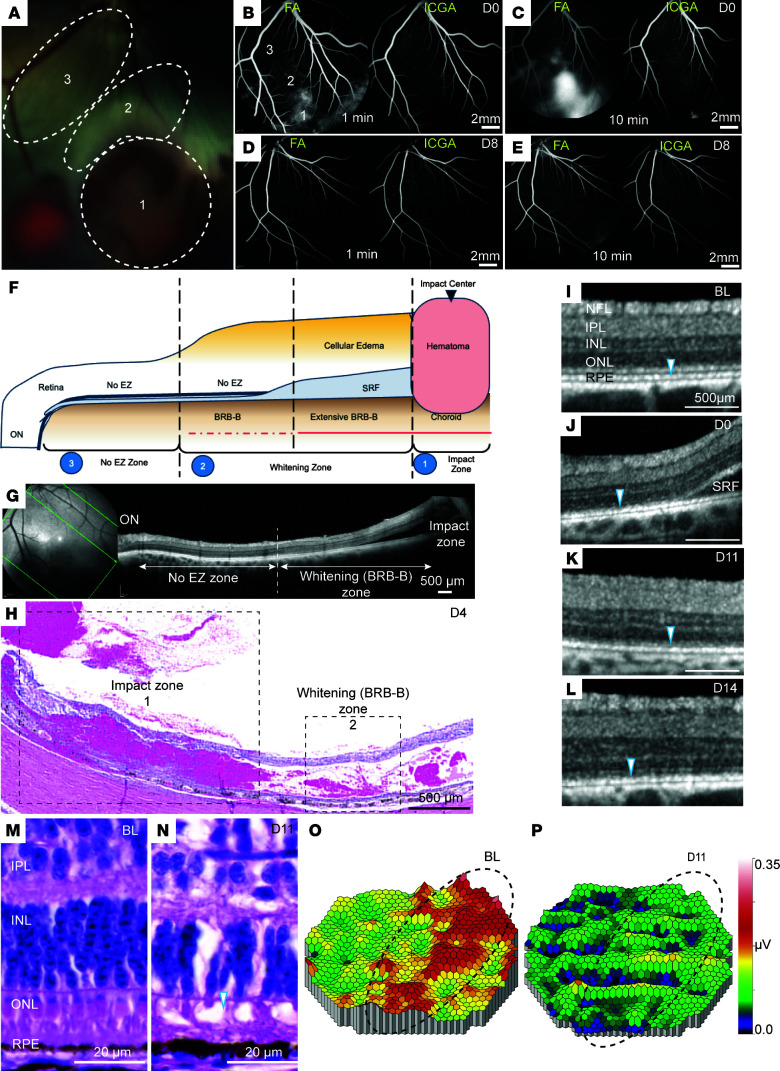
Short-term evaluation of pig eyes with CR injury. (**A**) High magnification of color fundus photograph of CR-injured eye showing: 1, preretinal hemorrhage at the impact site; 2, the whitening zone; 3, the adjacent visual streak. (**B**–**E**) FA images (left panels) show fluorescein dye leakage in early phase (1 minute) on day 0 (**B**) and late phase (10 minutes) on day 0 after injury (**C**), but not on day 8 after injury (**D**, early phase; **E**, late phase). ICGA images (right panels) show no dye leakage on day 0 after injury (**B**, early phase; **C**, late phase) and day 8 (**D**, early phase; **E**, late phase). Scale bars: 2 mm. (**F**) Schematic depicting the 3 distinct zones seen on color fundus and OCT images: impact zone showing hematoma (zone 1); whitening zone with extensive outer blood retina barrier (oBRB) damage and subretinal fluid (SRF) accumulation (zone 2); and zone with ellipsoid zone (EZ) disruption (zone 3). (**G** and **H**) OCT (**G**) and H&E staining (**H**) depicting the 3 zones described in **F**. Scale bars: 500 μm. (**I**–**L**) Higher-magnification OCT images: at baseline (BL) showing the ellipsoid zone (arrowhead, **I**); SRF accumulation on day 0 after CR injury (**J**); and fluid resorption by day 11 but missing ellipsoid zone (arrowhead, **K**), which persists on day 14 (**L**). Scale bars: 500 μm. (**M** and **N**) H&E section depicting healthy retina at baseline (BL) (**M**), and disruptions of ONL and photoreceptor outer segments (arrowhead) 11 days after CR injury (**N**). Scale bars: 20 μm. (**O** and **P**) mfERG signal heatmap at baseline (BL) (**O**) and day 11 (**P**) after injury showing the visual streak (vs) (dotted circle) and surrounding areas retina light response. Nine eyes were used for short-term evaluation of CR injury. NFL, nerve fiber layer; IPL, inner plexiform layer; INL, inner nuclear layer; ONL, outer nuclear layer; RPE, retinal pigment epithelium.

Based on our ex vivo analysis, we began testing on living pig eyes with projectile impact speed of 35.7 m/s. Five pigs were enrolled in this study. In all cases, fundus examination immediately after injury revealed the presence of preretinal hemorrhage at the area of impact (zone 1) and retinal whitening adjacent to it (zone 2) (Figure 3A). There was extensive outer blood-retinal barrier (oBRB) breakdown at zone 1 extending into zone 2, as evidenced by fluorescein leakage within a minute after dye injection continuing until the 10-minute evaluation time point (Figure 3, B and C, left). ICGA changes were minimal, suggesting no disruptions in retinal or deeper choroidal vessels (Figure 3, B and C, right). Fluorescein leakage subsided within 8–11 days after injury, suggesting a transient disruption of the oBRB with no additional changes in ICGA (Figure 3, D and E). OCT examination immediately after the injury revealed retinal detachment and subretinal fluid accumulation at the site of impact extending into zone 2 (Figure 3, F and G). OCT analysis also revealed extensive ellipsoid zone disruption extending from zone 2 into the visual streak (zone 3) (Figure 3, F and G). Quantification of OCT data revealed a range of sizes for different zones, illustrated in Figure 3F (zone 1: 2.0–4.0 mm; zone 2: 3.0–8.0 mm; zone 3: 2.0–8.0 mm). These findings were confirmed by histological analysis of the cadaveric pig eyes 4 days after the CR injury, revealing preretinal hemorrhage and extensive retinal damage with retinal atrophy in zone 1, thinning of outer retina and layer disorganization in zone 2, and photoreceptor disruption extending away from this area (Figure 3H). Follow-up by OCT confirmed FA/ICGA findings revealing subretinal fluid accumulation seen at day 0 (Figure 3, I and J) that was resolved in the first 2 weeks (Figure 3K). Interestingly, disruptions of the ellipsoid zone seen on day 0 persisted beyond day 14 (Figure 3, J–L). These findings were further confirmed by histological analysis of the retina (Figure 3, M and N), where photoreceptor outer segment shortening and ONL thinning and disruptions were evident. mfERG analysis showed reduced signal in the visual streak, confirming functional defects in cone photoreceptors (Figure 3, O and P, and Supplemental Figure 4). Overall, our data suggest that with impact speed up to 35.7 m/s, there was an acute oBRB breakdown that recovered by day 11, while the ellipsoid zone disruptions persisted.

### Long-term photoreceptor damage in PAD-induced CR.

**Figure 4:**
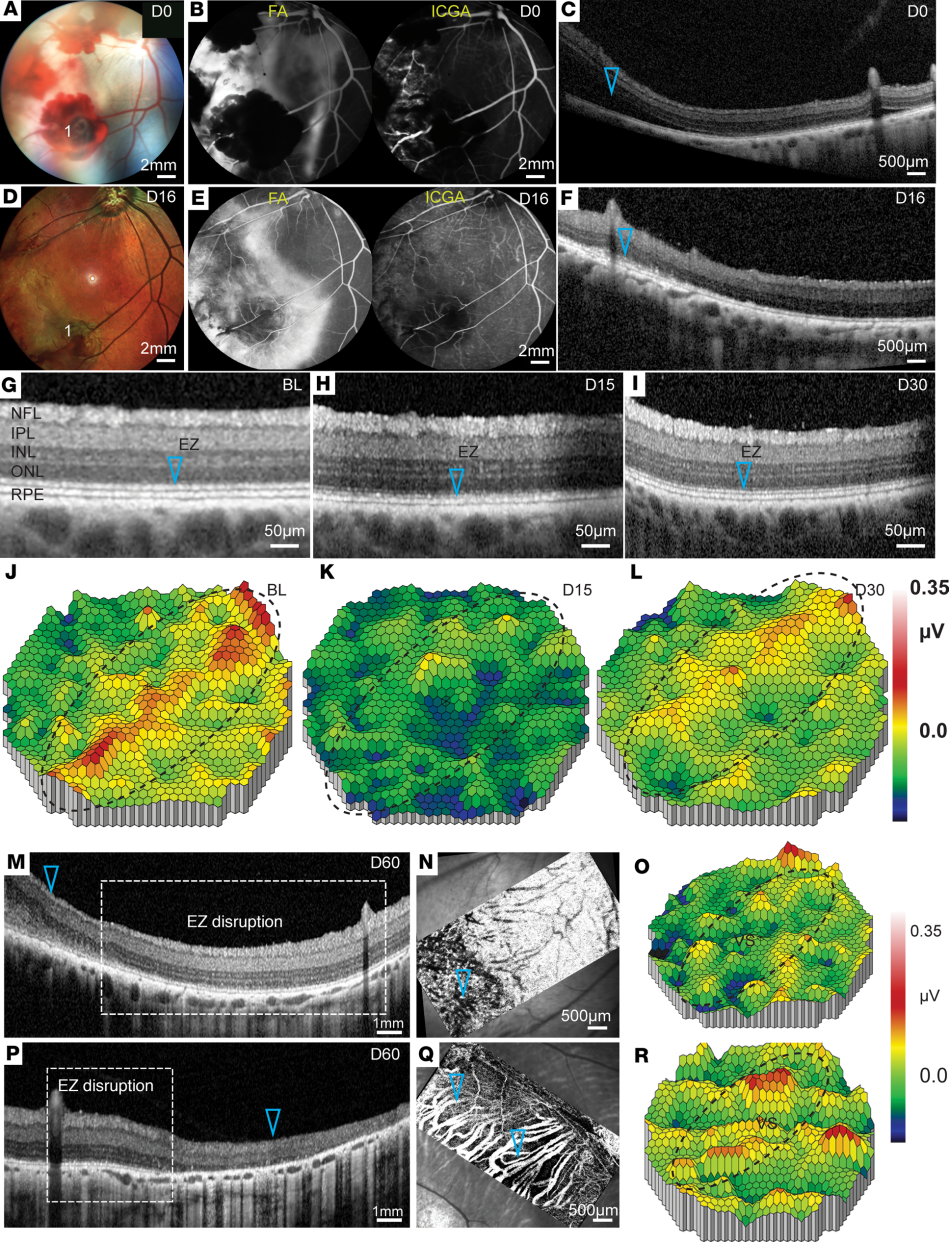
CR injury recovery in the long-term evaluation. (**A**–**F**) Color fundus (**A** and **D**), late-phase (10 minutes) FA (**B** and **E**, left panels), ICGA (**B** and **E**, right panels), and OCT (**C** and **F**) of post-injury eyes on day 0 (**A**–**C**) and day 16 (**D**–**F**) of evaluation of the same eye. “1” marks the area of impact showing preretinal hemorrhage. Whitened area in color fundus images corresponds to subretinal fluid accumulation on day 0, which is resolved by day 16 (arrowheads in **C** and **F**). Scale bars: 2 mm in **A**, **B**, **D**, and **E**; 500 μm in **C** and **F**. (**G**–**L**) OCT (**G**–**I**) and mfERG (**J**–**L**) analysis shows recovery of ellipsoid zone (EZ) (compare arrowheads in **G**–**I**) and recovery of mfERG signal in the visual streak (dashed ovals, **J**–**L**) by day 30 in eyes injured with a projectile speed of 35.7 m/s. Scale bars: 50 μm. (**M**–**R**) Comparative analysis at 60 days after injury using OCT (**M** and **P**), OCT angiography (**N** and **Q**), and mfERG (**O** and **R**) of 2 eyes injured with a projectile speed of 39.5 m/s highlights variability in damage to the outer retina and to the ellipsoid zone (arrowheads in **M** and **P**) and to the choriocapillaris (arrowheads in **N** and **Q**), and the variable signal in the visual streak (VS; dashed ovals) (**O** and **R**). Scale bars: 1 mm in **M** and **P**, 500 μm in **N** and **Q**. Seven eyes were used for this evaluation.

Short-term evaluation of the CR injury provided findings that were consistent with the patient data in terms of specificity of damage to photoreceptors (7, 38–43). Short-term evaluation also revealed acute oBRB damage not previously described for CR patients. With the goal of developing a suitable large-animal model for testing potential therapies, next we set out to determine whether PAD-induced CR injury at a projectile speed of 35.7 m/s persists in the longer term. Five pigs were enrolled in this part of the study and evaluated for up to 60 days after injury. As seen in the short-term studies, in all cases there was preretinal hemorrhage (zone 1) and retinal whitening — likely associated with an acute oBRB breakdown, which progressively healed by 16 days, as confirmed by fluorescein angiographs (Figure 4, A, B, D, and E). ICGA changes continued to be unremarkable by day 16 of evaluation, suggesting no damage to retinal or choroidal vessels (Figure 4, B and E). OCT analysis confirmed subretinal edema on day 0 in zone 2, which reabsorbed by day 16 (Figure 4, C and F). Surprisingly, the ellipsoid zone disruption evident in higher magnifications at day 15 recovered by day 30 (compare Figure 4G with Figure 4, H and I). This transient structural defect and its recovery were corroborated by initial loss and subsequent recovery of the mfERG signal, measured over the visual streak area (Figure 4, J–L). Because of this finding, we decided to increase the projectile impact speed to 39.5 m/s. Expectedly, higher speed caused deeper impact that was evident even at the 60-day follow-up, and there was no recovery of the ellipsoid zone on OCT (Figure 4, M and P). However, this high impact led to higher variability in structural damage to the retina with larger areas of retinal atrophy and relatively smaller ellipsoid zone disruption areas as assessed by OCT (compare Figure 4M with Figure 4P), variable non-perfusion of the choriocapillaris as seen by OCTA (compare Figure 4N with Figure 4Q), and variable functional changes in the visual streak as seen by mfERG (compare Figure 4O with Figure 4R). This variability, combined with the evidence of collateral damage to the posterior lens capsule and anterior segment (Supplemental Figure 5), prompted us to seek an alternative impact method to generate more reproducible, long-term, and specific injury to the photoreceptors without extensive anterior segment collateral damage.

### Scleral patch improves reproducibility of the CR injury.

**Figure 5:**
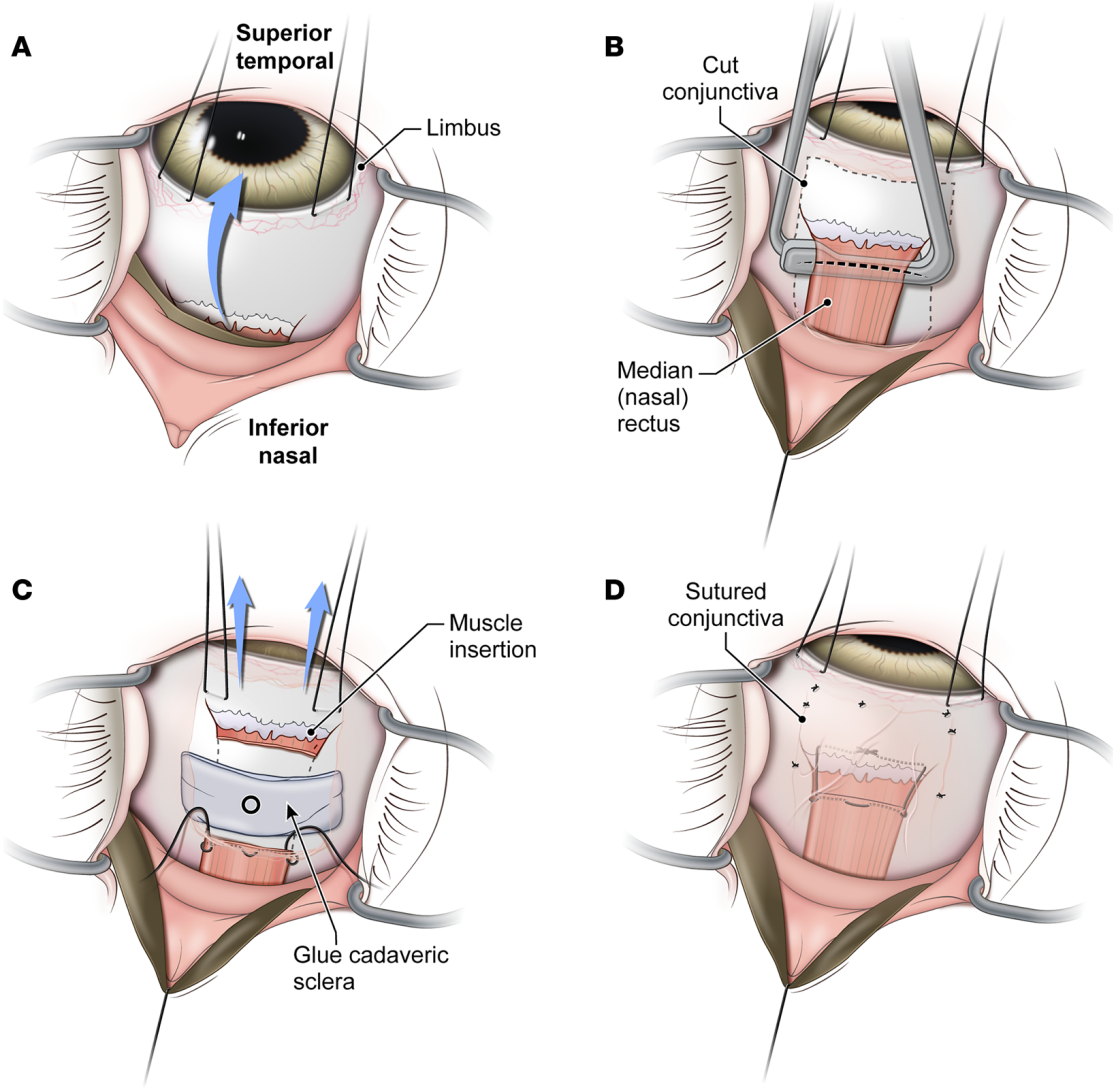
Surgical technique for cadaveric scleral patch placement. (**A**) Nasal sclera is exposed using limbal traction sutures. (**B**) The nasal conjunctiva is incised to expose and isolate the medial rectus muscle using hooks; dashed line marks the planned muscle transection site. (**C**) Scleral traction sutures are used to increase exposure of nasal sclera. A piece of cadaveric sclera is temporarily glued to the sclera, and the area of impact is marked (black circle). (**D**) After impact, the cadaveric sclera is removed, median rectus muscle is sutured back to its insertion, and conjunctiva is replaced and sutured (also see Supplemental Figure 6).

During post hoc analysis of evaluated eyes and based on literature evidence of variable scleral thickness in pigs, we asked whether we could control variability in injury extent by temporarily adding a scleral patch to the injury location (44). We used a commercially available cadaveric human scleral patch and glued it to the posterior sclera by disinsertion of the median rectus and release of the limbal traction (see Methods for surgery details). After the impact, the cadaveric scleral patch was removed, and the median rectus was resutured back to the sclera (Figure 5, A–D, and Supplemental Figure 6).

**Figure 6:**
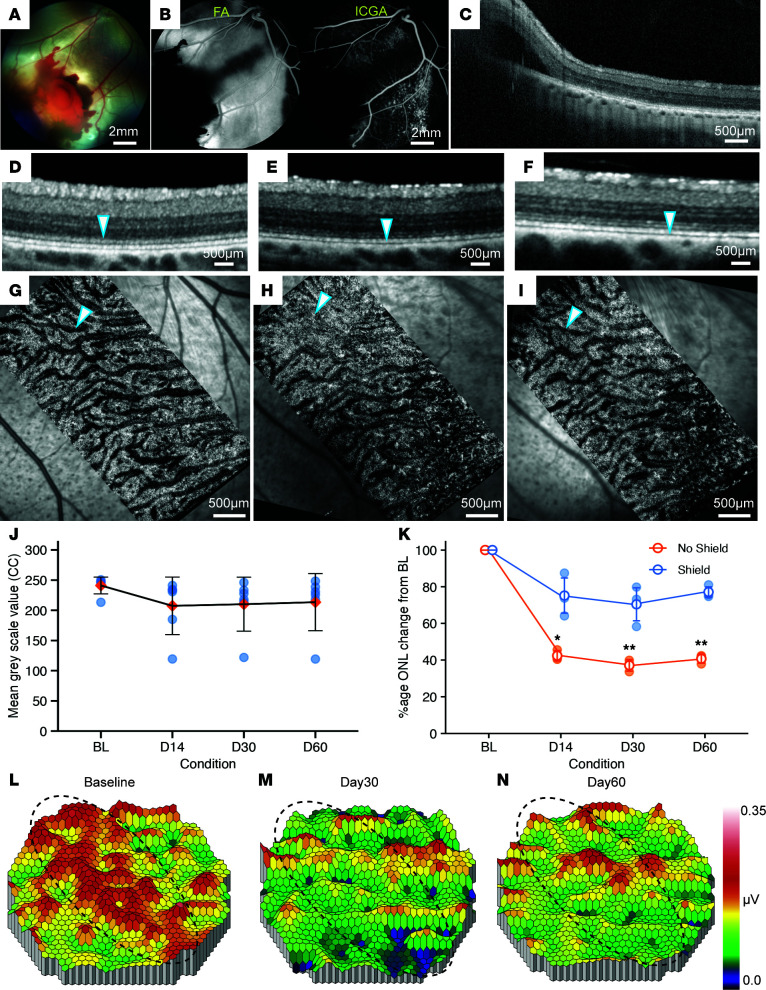
Long-term evaluation of CR injury induced with a cadaveric scleral patch support. (**A**) Color fundus image shows the area of impact with preretinal hemorrhage and the surrounding area of retinal whitening. (**B**) Late-phase (10 minutes) FA and ICGA show blocked fluorescence signal due to hemorrhage. Scale bars: 2 mm. (**C**) OCT showing subretinal fluid corresponding to the whitening area in **A**. Scale bar: 500 μm. (**D**–**I**) OCT images (**D**–**F**) and corresponding OCTA images (**G**–**I**) depicting the presence of ellipsoid zone and choriocapillaris, respectively, at baseline (**D**, arrowhead) and clear absence of ellipsoid zone at 30 days (**E**, arrowhead) and 60 days (**F**, arrowhead) after injury. Minimal changes are seen in choriocapillaris (compare **G**–**I**, arrowheads). Scale bars: 500 μm. (**J**) Median grayscale values intensity graph of OCTA signal intensity up to 60 days after CR injury. Results were analyzed using 1-way ANOVA. (**K**) Graph showing ONL thickness at baseline and 15, 30, and 60 days after projectile impact at 39.5 m/s on eyes with no scleral patch versus with scleral patch impacts. Data are presented as a percentage of average thickness of the same location at baseline. ANOVA (Friedman test compared with baseline) was used for statistical analysis. *P < 0.05, **P < 0.01. (**L**–**N**) mfERG heatmaps at baseline (**L**) and 30 days (**M**) and 60 days (**N**) after CR injury depicting changes in mfERG sensitivity throughout the evaluation time. Visual streak is highlighted by dashed ovals. Four eyes were used for this evaluation.

Four pigs were subjected to a projectile impact at a speed of 39.5 m/s and monitored for up to 60 days after injury. As seen with injury without the scleral patch, preretinal hemorrhage (zone 1) and retinal whitening (zone 2) seen immediately after the impact were also seen with the scleral patch, by color fundus photography (Figure 6A). FA confirmed that retinal whitening was caused by oBRB disruption (Figure 6B, left). As with the CR injury without the scleral patch, ICGA showed no signal, suggesting no damage to the choroidal vessels (Figure 6B, right). Edema seen on day 0 by OCT analysis resolved by day 30, suggesting that the oBRB heals by this time (Figure 6, C–E). In contrast, the ellipsoid zone disruption continued beyond day 30 until the day 60 evaluation time point (Figure 6, D–F). OCTA revealed a non-statistically-significant decrease in choriocapillaris density in the first 15 days after injury, with partial recovery by 60 days (Figure 6, G–J).

To confirm reproducibility of this approach, we compared OCT data across 4 eyes. As expected, by day 60, edema seen after projectile impact was reabsorbed (Supplemental Figure 7). The area of the impact showed noticeable retinal degeneration, and the adjacent area showed progressively improving retinal thickness with consistently missing ellipsoid zone (Supplemental Figure 7). To quantify changes seen in OCT, we performed segmentation of retinal layers using our recently published artificial intelligence–based algorithm for OCT segmentation (34). Segmentation analysis confirmed that use of the scleral patch resulted in a milder (~20%) reduction in ONL thicknesses as compared with injury without the patch (~50% reduction in ONL thickness) (Figure 6K). mfERG heatmap analysis showed a corresponding decrease in signal, persisting up to 60 days (Figure 6, L–N), consistent with longer-term photoreceptor damage. Our results indicate that the addition of a scleral patch to the impact area generated reproducible visual streak lesions and yielded a model that allows better understanding of human CR injuries and will help develop effective therapies for photoreceptor degeneration.

### Histological evaluation of PAD-induced CR injury to the retina.

**Figure 7:**
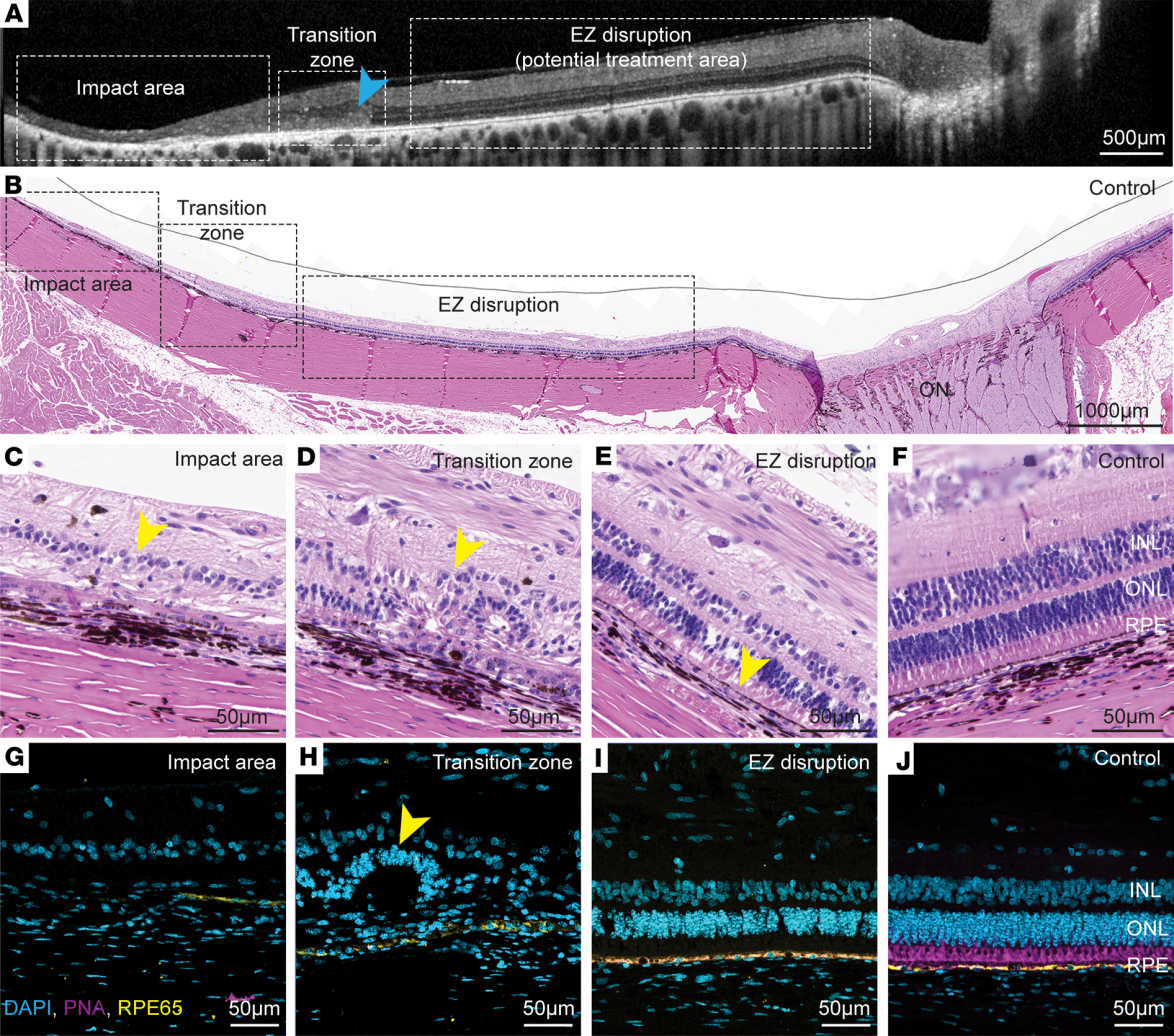
Histological analysis of CR model porcine eyes. (**A** and **B**) Terminal point OCT (**A**; arrowhead marks the transition zone) and corresponding H&E-stained section (**B**) showing the impact area with complete retinal atrophy, the transition zone with significant outer retina damage, and an area with ellipsoid zone (EZ) disruption. Scale bars: 500 μm in **A**, 1,000 μm in **B**. (**C**–**J**) Higher-magnification views of the impact area (**C** and **G**) showing complete retinal atrophy (arrowhead), the transition zone (**D** and **H**) showing outer and inner retina layer degeneration (arrowheads), (**E** and **I**) showing degenerated photoreceptor outer segments and EZ disruption (arrowhead) with relatively preserved photoreceptor outer nuclear layer (ONL), and control retina (**F** and **J**), stained with H&E (**C**–**F**) or stained for cone photoreceptors (PNA, magenta), RPE (RPE65, yellow), and nuclei (DAPI, cyan) (**G**–**J**). Scale bars: 50 μm.

To further evaluate the impact of CR injury on porcine retina, we performed histological analysis of eyes at the end of 60 days of longitudinal live imaging. Consistent with the OCT data (Figure 7A), histological (Figure 7, B–F) and immunostaining analysis provides qualitative analysis of 3 distinct areas of injury (Figure 7, G–J): the impact zone, a transition zone, and the ellipsoid zone disruption area. In the impact zone, H&E staining showed complete atrophy of both inner and outer retinal layers (compare Figure 7, B, C, and F); immunostaining further confirmed missing signals for cone photoreceptors (peanut agglutinin [PNA]) and retinal pigment epithelium (RPE) (RPE65) (compare Figure 7, G–J). In the transition zone, H&E and immunostaining showed partial preservation of the inner retina and rosette-like structures with disruptions in outer retinal layers including the RPE, photoreceptor outer segment (POS), and ONL; and barely visible RPE layer with faint RPE65 immunostaining and no PNA signal, suggesting missing cone photoreceptor outer segments (compare Figure 7, B, D, and F, with Figure 7, H and J). The ellipsoid zone disruption area had a thinned ONL lacking PNA signal but relatively intact RPE and inner nuclear layer (compare Figure 7, B, E, and F, with Figure 7, I and J). Overall, this histological analysis (H&E and immunostaining) corroborated our in vivo structural and functional evaluations, confirming the loss of photoreceptors for the evaluation period of up to 60 days in PAD-induced closed-globe CR injury.

## Discussion

We report a reproducible large-animal model of posttraumatic photoreceptor degeneration that mimics macular injury seen in patients with closed-globe CR injuries. Injury in this porcine model recapitulates several features of the human macular CR injury, including (a) involvement of the macula-equivalent visual streak in pigs; (b) a transient oBRB breakdown; (c) a transient subretinal fluid accumulation that resolves by 7–14 days; (d) damage limited to photoreceptor outer segments and recovered by 30 days after injury with lower-energy projectile impacts (at or below 38 m/s projectile speed); and (e) persistent absence of the ellipsoid zone and ONL thinning for the entire evaluation period of 60 days, suggesting permanent damage, following injury caused by projectile impact speed of 39.5 m/s.

Commotio retinae was originally named Berlin’s edema and was described as retinal whitening involving loss or disruption of photoreceptor outer segments (28, 45). Previous reports of oBRB breakdown in closed-globe injuries have been contradictory. In a series of 21 patients evaluated with FA and ICGA, findings varied (46). More specifically, fluorescein dye leakage occurred in 9 of 21 eyes, and a “salt and pepper” appearance was observed in one, which the authors felt indicated a more severe injury (though visual acuity was not reported in these cases). In some cases, the early increase in choriocapillaris permeability developed into choriocapillaris vascular occlusion by day 4. In cases where evaluated, abnormal FA and ICGA showed delayed filling of the choriocapillaris (20, 22–24, 29, 45, 46). This finding led the authors to speculate that in severe CR, occlusion of the choriocapillaris causes outer retinal ischemia, impairing recovery. More recent studies report subretinal fluid only in the most severe injuries (29). In comparison with these previous reports, in our model, oBRB breakdown and subretinal fluid were seen in all cases, including those that did not recover defects in outer retina structure and function. It is likely that previous reports, in which patients were assessed at variable times after injury, transient and localized (outside the posterior pole) oBRB breakdown may have been missed. Another possible reason for this discrepancy may have to do with location of injury, which is variable in human blunt-force injuries. Our ability to generate a reproducible injury, combined with our subsequent systematic and comprehensive analysis, leads us to speculate that oBRB breakdown may be a common, if not a universal, feature of CR injuries, and that the loss of the choriocapillaris does not directly correlate with outer retina damage or its recovery. While retinal detachment in the context of severe ocular trauma is a concern, acute subretinal fluid accumulation after blunt trauma at the site of commotio or sclopetaria retinae may be serous, as indicated by previous studies (46, 47). But the oBRB breakdown seems to resolve with time in most cases. This finding is consistent with published clinical reports (20), supporting the transient nature of the oBRB breakdown.

The impact sites and speed reported in previous animal models of CR (47) are variable, but in general, higher-speed impacts of low-weight projectiles were shown to cause persistent and reproducible CR injury as compared with lower-speed impacts from heavy projectiles that caused more damage at the impact site and the neighboring retina (16, 19, 21, 27). Higher projectile speed produces desired damage in our model; however, the site of injury was critical in obtaining reproducible and retina-specific damage. We used a scleral injury site because corneal impacts with energies sufficient to induce CR also damaged the anterior segment — inducing corneal edema, cataract, iridodialysis, and hyphema. These anterior segment disruptions preclude proper evaluation of the retinal damage using clinically relevant imaging modalities such as OCT, OCTA, mfERG, and FA/ICGA. Scleral impacts not only avoid anterior segment disruption but also produce the same ultrastructural features of CR injury seen in patients. Putting these findings together, scleral impact–induced closed-globe CR injury provides a more clinically relevant and reproducible animal model for better understanding CR etiology and for testing injury-specific therapeutic approaches.

At optimized projectile speeds, photoreceptor outer segment damage was visible after 1 week of injury and continued for the 2-month evaluation period. Our OCT findings are consistent with previous reports (38–42, 48); namely, with projectile speed of 37.5 m/s or less, there was an initial increase in reflectivity of the inner/outer segment ellipsoid zone with disappearance of the thin hyporeflective optical space. In these cases, OCT changes recovered over time. With projectile speed of 39.5 m/s, OCT revealed disruption of the inner and outer segment layers with partial atrophy of the ONL, and no recovery of the ellipsoid zone and the ONL during the evaluation period of 60 days. These results are consistent with Chen et al., who found that foveal thickness and grade of outer retinal atrophy were predictors of final visual outcome (8, 41). The ellipsoid zone and ONL thinning in the macular area may therefore also help predict which patients could benefit from what kind of therapies.

Our mfERG findings are also in agreement with previous reports of transient decrease in mfERG amplitude with lower projectile speed (43, 49). With projectile speed of 37.5 m/s, the mfERG amplitude was significantly reduced after the injury but recovered over a 30-day period, while, with projectile speed of 39.5 m/s, the mfERG amplitude remained low for the entire evaluation period of 60 days. Similarly to Mansour et al. (50), we report the presence of preretinal, retinal, and subretinal hemorrhage around the area of impact as seen by fundus imaging and histology. The preretinal hemorrhage being sub-hyaloid could explain its rapid reabsorption in our cases (11). Furthermore, we provide, to our knowledge, the first immunofluorescence findings in a CR injury, confirming that photoreceptor degeneration occurs while RPE cells remain viable 60 days after injury. Overall, our model confirms what has been reported in previous rodent models of CR injury but also furthers the field with findings that are consistent with the human injury.

Our model has several advantages over previous CR models: (a) the pig eye is similar in size and retinal structure to the human eye (32); (b) the pig visual streak is cone rich, like the human macula; (c) using a posterior scleral injury approach, we were able to preserve the health and transparency of the cornea and the lens, allowing us to perform longitudinal structural and functional assessment of the retina using clinically relevant imaging modalities; and (d) retinal analysis led us to discover 3 discrete areas: the impact zone, the transition zone — an area adjacent to the direct impact site where the damage to the ONL and ellipsoid zone was extensive — and the ellipsoid zone disruption area with photoreceptor-specific damage to the ellipsoid zone and the ONL. One limitation of our model is that it may not fully capture the disease-associated pathophysiology of photoreceptor-specific retinal degeneration. Therefore, it will be important to also evaluate photoreceptor transplants in disease models, such as the P23H rhodopsin mutation model of retinitis pigmentosa (51). Nonetheless, our model represents a valuable platform for advancing the development of vitreoretinal surgical techniques, instrumentation, and potential therapies for photoreceptor degeneration.

## Methods

### Sex as a biological variable.

Sex was not considered as a biological variable. CR injury is not anticipated to be different between males (castrated or non-castrated) and females. Castrated males were used for this study because only they (not non-castrated males or females) are amenable to social housing, allowing an enriched social environment for the animals (52).

### Study design.

All animals received a baseline examination prior to the CR injury, including OCT, OCTA, FA/ICGA, and mfERG. The same examinations were repeated immediately after the CR injury and at around 7–11, 15, 30, and 60 days after. Because of logistics of animal handling, different animals could not be followed on the same day. Hence, they were followed within a window of a few days. Animals were euthanized at different time points up to 60 days, and the eyes were collected for histology (H&E, Masson’s, and immunofluorescence evaluation).

### Animal care and procedures.

Yorkshire and Yucatan minipigs from Premier BioSource/S&S Farms and Sinclair Research were enrolled in the study. Since pig eyes are fully developed by 6 months, the only limitation for long-term evaluations between both breeds was the rate of growth, which was slower in the Yucatan breed. Animals (castrated males, 35–45 kg) were housed in climate control rooms illuminated at 25–37 lux with a 12-hours-on cycle and wood shavings on the floor. Food was provided twice a day, and water was offered ab libitum. For imaging and CR injury, pigs were anesthetized, intubated, and maintained on a pressure-controlled ventilator, as previously described (52). Pigs were positioned in custom cradles and water, and air-warming blankets were used to maintain the body temperature. Blood pressure, heart rate, blood oxygenation, CO_2_, and temperature were monitored continuously. Sodium chloride (0.9% sodium chloride injection, USP; Hospira) or Lactated Ringer’s (Lactated Ringer’s injection, USP; ICU Medical) solutions were administered throughout the procedure at an average flow rate of 10 mL/kg/h. Pupils were dilated with tropicamide 1% (Tropicamide Ophthalmic solution 1%, USP; Akorn or Sandoz) and phenylephrine 10% (phenylephrine hydrochloride ophthalmic drops 10%, USP; Paragon Biotech). During image acquisition and CR injury, rocuronium (2–3 mg/kg, i.v., rocuronium bromide injection 10 mg/mL, USP; XGen) was administered and repeated as needed for relaxation of the extraocular muscles. After CR injury, 0.4 mL subconjunctival cefazolin (330 mg/mL) was administered. Upon completion of procedures, an ophthalmic ointment (neomycin and polymyxin B sulfates ophthalmic ointment, USP; Bausch & Lomb) was applied on the corneal surface. Ketoprofen (3 mg/kg, i.m., Ketofen, 100 mg/mL; Zoetis) was administered to reduce pain related to the procedures performed. Fluorescein and indocyanine green were administered intravenously. In preparation for enucleation, pigs were anesthetized using the protocol outlined above. Animals were euthanized by administration of B-euthanasia i.v. 1 mL per 10 lbs of body weight (Euthanasia Solution, VetOne), and eyes were enucleated. The animal’s heart rate, blood pressure, and respiration were monitored to confirm euthanasia.

### CT scan and 3D skull reconstruction.

CT scans of the pig head were performed in the Section on Cognitive Neurophysiology and Imaging Laboratory of Neuropsychology (National Institute of Mental Health). An Epica Vimago HU Veterinary CT Scanner was used (ARO Systems) to image anesthetized male Yucatan minipigs. Anatomical Worldwide (https://www.anatomicalworldwide.com/) company was contracted to use Digital Imaging and Communications in Medicine (DICOM) set images from the CT scan to design and 3D-print the porcine skull. Ballistic gel was purchased from EnvironMolds ArtMolds. It consisted of a 10% non-gelatin clear synthetic gel, which is clear as glass, odorless, reusable, and temperature stable (up to 240°F) and mimics human tissue elasticity. The ballistic gel covered the skull and was introduced into the orbit of the 3D-printed porcine model to mimic as much as possible the consistency and resistance of the orbital tissue at the time of projectile impact.

### Pressure application device.

To deliver a small spherical plastic polyoxymethylene (POM) projectile (ball ~12 mm, 0.75 g; VXB Bearings), the PAD consisted of a simple closed pneumatic system, using a compressed gas cylinder to generate precisely measured pressure, triggered by a solenoid-actuated valve (SMC Pneumatics VQ31A1-5YH-C12 4/5 port solenoid valve from Automatic Distribution Inc., Hartfield, Pennsylvania) with a bead loading port and an aiming laser beam for precise delivery to the specific area (Figure 1A and Supplemental Figure 1A). The device was connected to a nitrogen tank and a pressure manometer allowing precise control of the exerted pressure (measured in pounds per square inch [psi]). A remote control triggered the projectile (Figure 1A).

The exit speed of the projectile was measured using a laser photogate and the time-transit method. Collimated laser light (1054, Adafruit) crosses the path of the launch tube’s diameter where it illuminates a photodiode (FDS100, Thorlabs) masked with a 1.0 mm–wide aperture. A transimpedance amplifier (MCP6022, Microchip) converts the photocurrent to a voltage for readout (Supplemental Figure 1A). A microcontroller and oscilloscope were used to measure the transit time (*t*) that the projectile blocked the laser during transit. The diameter (*d*) of the projectile divided by time (*t*) yielded the projectile’s exit speed (*s* = *d*/*t* in meters per second).

### Commotio retinae injury.

Closed-globe commotio retinae (CR) injury was created under general anesthesia, as described above. The nasal sclera was exposed using 2 limbal traction sutures (6-0 silk, black braided, MANI). A perilimbal nasal peritomy with 2 radial incisions was made in the conjunctiva to expose the medial rectus. Using a muscle hook, the muscle was isolated, its insertion was highlighted with tissue marker (Viscot Medical LLC), and a double-armed suture (6-0 polyglycolic acid [PGA], violet braided, MANI) passed through the muscle close to the insertion before its disinsertion. Using binocular indirect ophthalmoscopy, the center of the visual streak was identified, and the area of intended impact overlying the visual streak was highlighted using a tissue marker. In later experiments, to allow for a more posterior impact (to minimize anterior segment damage) and to obtain more localized and reproducible damage, scleral traction sutures and a commercially available cadaveric human scleral patch ranging in thickness from 150 to 250 μm (Tutoplast processed sclera, Katena) glued to the nasal sclera (Figure 5 and Supplemental Figure 6) were used (Vetbond tissue adhesive, 3M). After retraction of conjunctiva, limbal traction sutures were used to expose the nasal sclera. The median rectus was identified, sutured, sectioned, and retracted. Scleral sutures were used to further expose the nasal sclera. The cadaveric human scleral patch was temporarily glued to the sclera below the median rectus insertion, and the impact area was highlighted. Once exposed, the PAD laser was aligned with the highlight, with the exit tube 2 inches from the sclera, and POM balls at different velocities were tested. After impact, the cadaveric sclera was removed, the median rectus sutured in its insertion, and the conjunctiva resutured in the limbus.

### Optical coherence tomography.

Optical coherence tomography (OCT) images were obtained using the SPECTRALIS Spectral-Domain OCT (HRA3, Heidelberg Engineering) instrument and recorded as previously described (52). During each imaging session, 3 OCT volumes were recorded for each eye: 1 radial scan (centered in the visual streak) and 2 raster scans. Raster scans were recorded parallel and perpendicular to the visual streak. To improve signal-to-noise ratio, speckle noise, and contrast, each scan was averaged over 19 ± 2 images with the automatic real-time tracking function. Radial and raster scan volumes consisted of up to 48 images and 217 images, respectively. To quantify the effect of the CR injury and the progression of damage, cross-sectional areas of retinal layers were recorded in equally sized OCT B-scans and compared over time on co-registered follow-up images. All OCT B-scans were exported in TIFF format using Heidelberg Eye Explorer 2 (HEYEX 2) software.

### OCT segmentation.

For segmentation analysis, 5 cross-sectional areas, evenly divided throughout the OCT volume, were analyzed based on our recently developed algorithm (34). The outer border of the visual streak was defined as the borders of the retinal arteries and veins that surround the visual streak. The 3 equal inner longitudinal lines were used for the analysis (Supplemental Figure 4). To ensure segmented areas were similar at all the time points, the eye tracking device was used in parallel raster scans. All OCT B-scans were manually segmented by an unbiased observer with no prior involvement in the study and the scan export process. Specifically, the inner and outer boundaries of the outer nuclear layer (ONL) were segmented. Annotations were constrained within the visual streak of the pig and outside of scarred regions.

### Segmentation data analysis.

All segmentation files were saved in JSON format. Subsequently, our recently developed MATLAB (MathWorks) scripts were used to calculate the axial thickness (34). Axial layer thickness was averaged for each B-scan and was normalized to the corresponding B-scan at baseline. A nonparametric repeated-measures ANOVA (Friedman test) was performed using GraphPad Prism version 9.5.0 for Windows (GraphPad Software).

### OCT angiography.

OCT angiography (OCTA) images were obtained using the SPECTRALIS Spectral-Domain (SD) OCT (Heidelberg Engineering) instrument. Each OCTA B-scan contained between 384 and 768 A-scans, and each OCTA volume contained between 256 and 512 B-scans. OCTA volumes were centered on specific regions of interest.

### OCTA analysis.

En face OCTA scans were exported from the HEYEX Heidelberg software in TIFF format. Choriocapillaris vasculature in each CR region was directly compared with choriocapillaris vasculature in baseline analysis of the same area. An experienced observer obtained the gray value from each en face scan, of the average binary pixel intensity, for as big an area as possible in both CR and baseline regions. All gray value calculations were completed using ImageJ (NIH). For each image, the gray value ratio CR was compared with baseline and was reported as a percentage change.

### Fluorescein and indocyanine green angiography.

Fluorescein angiography (FA) and indocyanine green angiography (ICGA) were obtained using the SD OCT system after intravenous injection of 1 mL fluorescein 10% solution (AK Fluor 10%, USP; Akorn) and 5 mg indocyanine green (Indocyanine Green 25 mg, USP; Diagnostic Green). A first-minute movie and 1-, 5-, and 10-minute frames were obtained after the injection.

### Multifocal electroretinography.

The Reti-map animal multifocal electroretinography (mfERG) system (Roland Consult) was used with a 2-channel bio-signal amplifier (stimulus frequency selection 10–100 Hz) to collect an array of 241 black-and-white hexagons at 10 μV over 30 to 40 degrees of the central visual field, thus allowing accurate evaluation of the pig visual streak (Supplemental Figure 5). An active contact lens electrode was placed on the cornea using a coupling gel (Genteal, Alcon Pharmaceutical). The electrode was connected to an amplifier, and a second electrode was connected to the “ground input” of the amplifier. The pupils were maximally dilated and centered within the ring of the corneal electrode. Recordings were performed under photopic conditions, thus excluding rod contributions to the signal, and ensuring a primarily cone-driven response (52). Considering the variability of the photopic response even within the same day, the Reti-map was set to average 3 scans for each selected area of the retina.

### Eye fixation and sectioning.

Cadaveric eyes were fixed immediately after injury, in 4% paraformaldehyde for four hours, and transferred to 1% in PBS until histological processing. Some eyes were dissected open immediately after injury for gross examination before fixation. After fixation, eyes were dissected open to identify area of interest and processed for histology processing.

Animals were euthanized, and the eyes were collected and processed for histological evaluations. Eyes were fixed for 8 days in 2% paraformaldehyde and 2% glutaraldehyde to maintain tissue morphology, then placed in 70% alcohol overnight and washed in running tap water for 24 hours.

During histological processing, retinal retraction on intact sclera was minimized with 15% alcohol. Absolute ethyl alcohol and water (1:15) mixture was used to pretreat the sections 15 minutes before they were transferred to tissue flotation bath and onto glass slides. The solution was freshly prepared (53).

Paraffin-embedded tissues were sectioned at 4 μm thickness using a Leica microtome (Leica Biosystems).

Cross sections contained all retinal layers from the ora serrata to the posterior pole. Sections were deparaffinized and stained with Harris’s hematoxylin and eosin (H&E), Y Phloxine B to counterstain H&E, or Masson’s trichrome (StatLab).

### Immunostaining of paraffin sections.

Deparaffinization was performed as previously described (54), followed by antigen retrieval in preheated citrate buffer (1×, pH 6.0) using a water bath for 15 minutes. Then primary antibody incubation was performed overnight at room temperature. The following primary antibodies were used: RPE65 mouse antibody (1:500; catalog ab175936, Abcam Inc.), and peanut agglutinin (PNA) (5 mg, 1:500; FL-1075-5, Vector Labs) conjugated with a 488 fluorophore. Secondary antibody Alexa Fluor 568 (1:300; Invitrogen, Thermo Fisher Scientific) was incubated for 30 minutes at room temperature. Sections were also stained with Hoechst 33342 (1:1,000; catalog 62249, Invitrogen, Thermo Fisher Scientific).

### Statistics.

For analysis of average velocities, GraphPad Prism version 9.5.0 was also used, and results were analyzed using 1-way ANOVA. A *P* value of less than 0.05 was considered significant.

### Study approval.

All animal procedures were performed in accordance with the guidelines of the Association for Research in Vision and Ophthalmology statement on the use of animals in ophthalmic and vision research. All animal procedures received prior approval from the National Eye Institute, NIH, Animal Care and Use Committee.

### Data availability.

Raw values are provided in the Supporting Data Values file. Segmented JSON files for OCT are available upon request.

## Author contributions

JA, IB, AM, MMC, FB, RG, MF, and JN developed and tested the CR porcine model. MJP, RS, DMG, KB, and RJB contributed to study design, data analysis, and manuscript writing. KB approved the manuscript.

## Funding support

This work is the result of Intramural Research Program NIH funding, in whole or in part, and is subject to the NIH Public Access Policy. Through acceptance of this federal funding, the NIH has been given a right to make the work publicly available in PubMed Central. The contributions of the NIH authors were made as part of their official duties as NIH federal employees, follow agency policy requirements, and are considered Works of the United States Government. However, the findings and conclusions presented in this paper are those of the authors and do not necessarily reflect the views of the NIH or the US Department of Health and Human Services.

- National Eye Institute (NEI) Intramural Research Program funds (to KB).
- Department of Defense grant W81XWH-20-1-0655 (to DMG and KB).
- Research to Prevent Blindness (to DMG).

## Supplementary Material

Supplemental data

Supplemental video 1

Supporting data values

## Version 1 01/13/2026

In-Press Preview

## Version 2 02/23/2026

Electronic publication
