# Supplementary material for: A porcine commotio retinae model for preclinical evaluation of posttraumatic photoreceptor degeneration
Source: JCI Insight. 2026 Jan 13;11(4):e192799. doi: 10.1172/jci.insight.192799 (PMC12956011; doi:10.1172/jci.insight.192799)
Supplement: Supplemental data [file jciinsight-11-192799-s081.pdf]

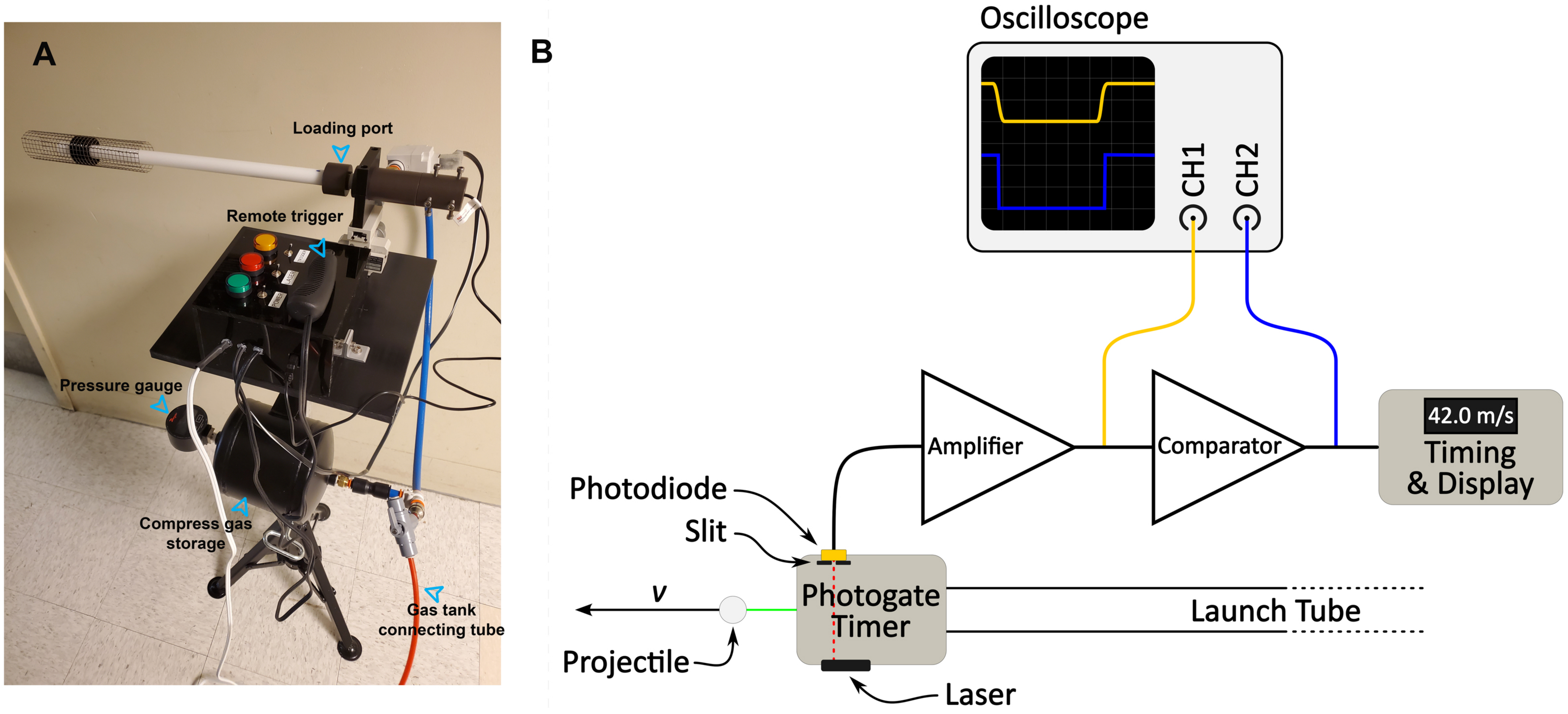
A
Loading port
Remote trigger
Pressure gauge
Compress gas storage
Gas tank connecting tube
B
Oscilloscope
CH1
CH2
Amplifier
Comparator
42.0 m/s
Timing & Display
Photodiode
Slit
v
Photogate Timer
Launch Tube
Projectile
Laser

Supplemental Fig. 1. Pressure application device (PAD) functioning. (A) Photograph of the PAD device. **(B)** Circuit for projectile speed measurement showing the laser beam that traverses the projectile launch tube and the narrow slit that masks the photodiode. The photodiode is connected to a high bandwidth transimpedance amplifier, comparator, and microcontroller circuit. The analog and digital signals are simultaneously monitored with an oscilloscope to verify accuracy of microcontroller timing measurements.

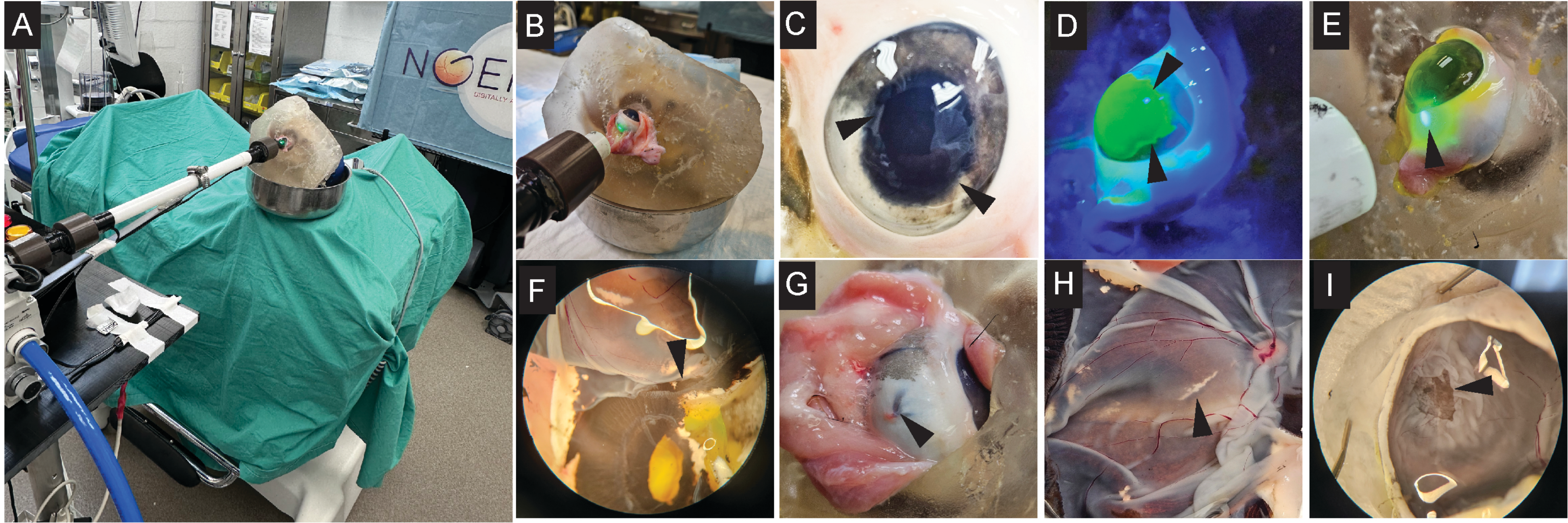
A
B
C
D
E
F
G
H
I

Supplementary Fig. 2. Ex vivo PAD evaluation. (A, B) Room set up **(A)** and higher magnification view **(B)** showing PAD aimed at a cadaveric pig eye in a 3D printed pig skull filled with the ballistic gel®. **(C)** Arrow heads point to epithelial displacement in the cornea highlighting corneal damage after a direct projectile impact. **(D)** Fluorescein dye stains damaged corneal cells (arrow heads) after a direct projectile impact. **(E)** Fluorescein dye stains peripheral epithelial corneal defects (arrowhead) after limbal impact of the projectile. **(F)** Arrowhead points to limbal impacts associated retinal dialysis. **(G-I)** Scleral impact at 48.14 m/s induced extensive scleral damage (**G**, arrow), and rupture in the choroid (**H**, arrow) and the retina (**I**, arrow).

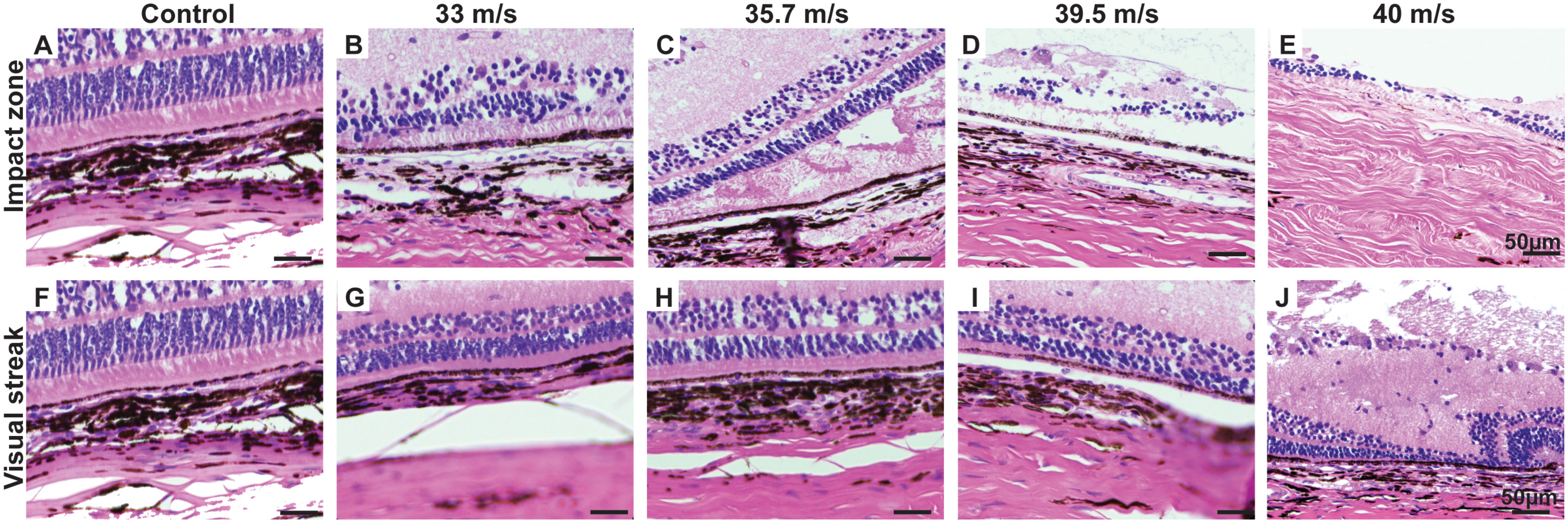
Control
33 m/s
35.7 m/s
39.5 m/s
40 m/s
Impact zone
Visual streak
A
B
C
D
E
F
G
H
I
J
50µm
50µm

Supplemental Fig. 3. Projectile speed optimization for the scleral impact. H&E stained images showing the extent of damage in the impact zone **(A-E)**, and the visual streak **(F-J)** and eyes hit with projectiles at different velocities – 15 PSI – 33 m/s **(B, G)**, 17 PSI – 35.7 m/s **(C, H)**, 19 PSI – 39.5 m/s **(D, I)**, and 25 PSI – 48m/s **(E, J)**.

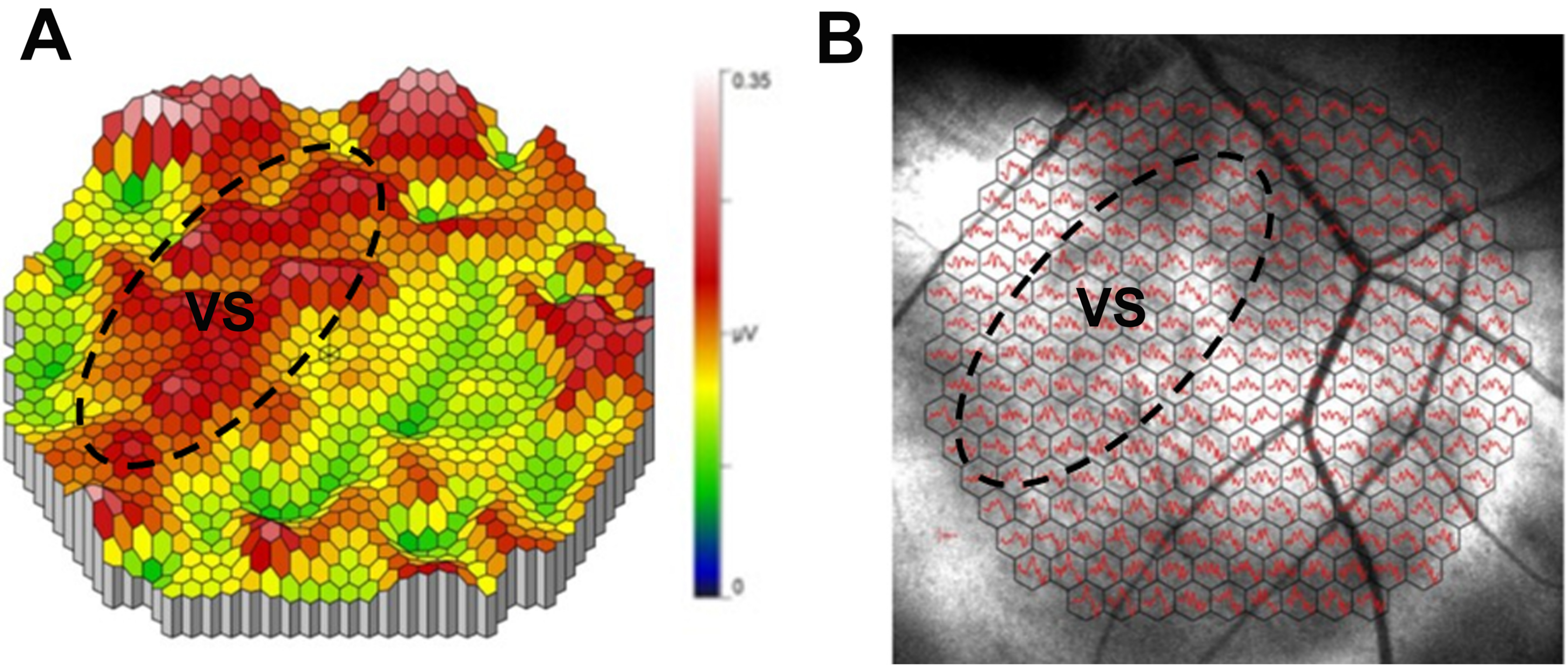
A
VS
0.35
µV
0
B
VS

Supplemental Fig. 4. mfERG protocol. **(A)** 3D heat map of peak 1 (P1) amplitudes and infrared fundus image **(B)** showing the location where light stimuli were projected on the retina. Average mfERG signal obtained per hexagon is shown. Higher amplitude peaks (represented in red and orange in the left panel) correlate with the high concentration of cones in the visual streak (VS) area (marked by dotted oval in both panels).

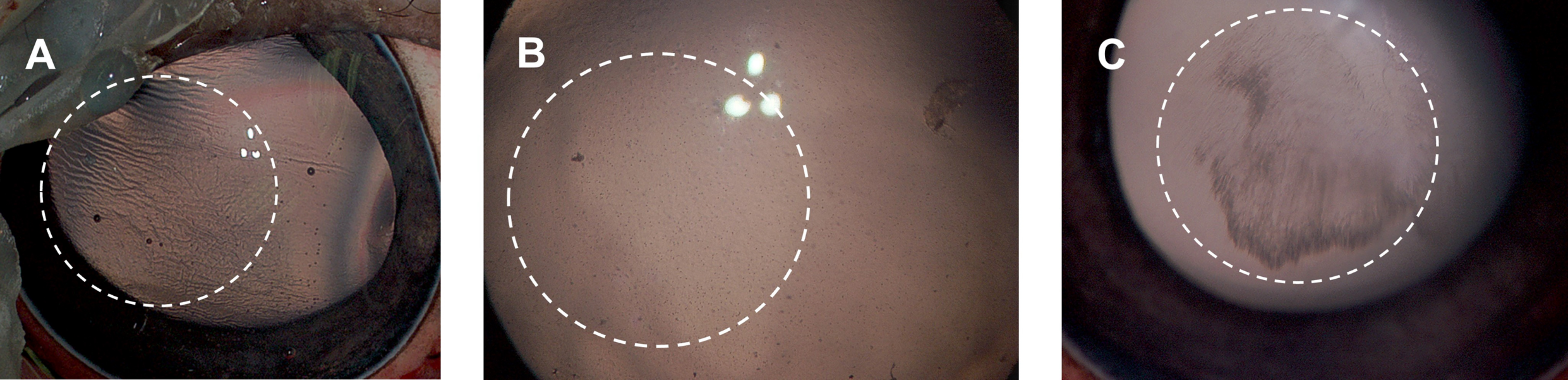
A
B
C

Supplementary Fig. 5. Anterior segment changes after the scleral impact. Examples of corneal folds (circle) **(A)**; anterior chamber cells (circle) **(B)**; posterior capsule tattoo (circle) secondary to pigment dispersion after impact shock wave **(C)**.

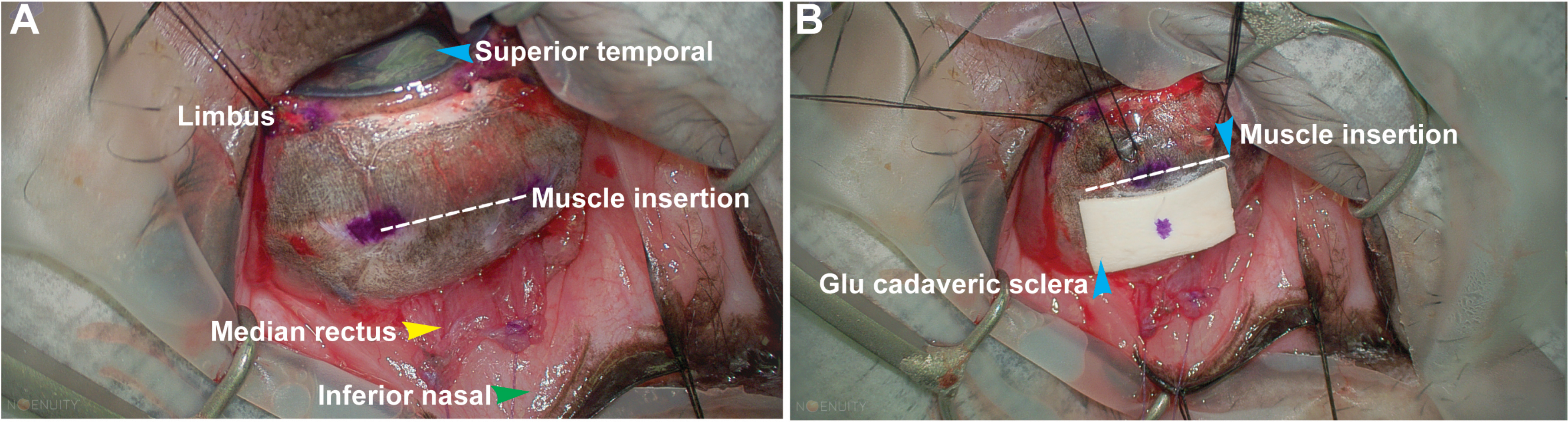
A
Superior temporal
Limbus
Muscle insertion
Median rectus
Inferior nasal
B
Muscle insertion
Glu cadaveric sclera

Supplementary Fig. 6. Surgical technique for nasal scleral impact using the cadaveric scleral patch. **(A)** Exposed nasal sclera after retracted conjunctiva. The area between superior temporal (blue arrow) and inferior nasal (green arrow) is the nasal sclera. Dotted line shows cut median rectus muscle and yellow arrow shows the retracted muscle. **(B)** Cadaveric human scleral patch (marked by blue arrowheads) is temporally glued to the nasal sclera below median rectus insertion (dotted lines) and impact location is marked (purple dot).

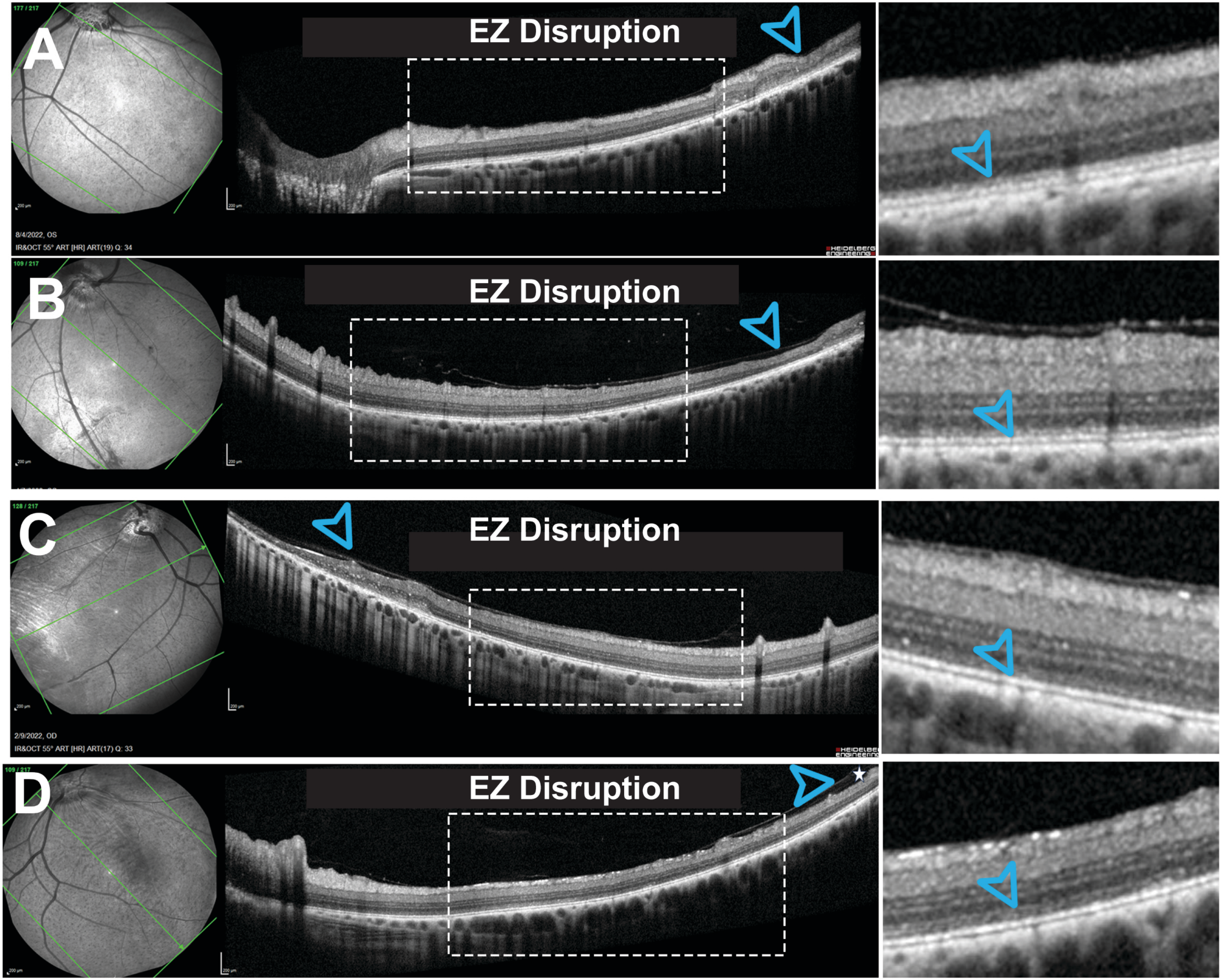
A
EZ Disruption
177 / 217
8/4/2022, OS
IR&OCT 55° ART [HR] ART(19) Q: 34
B
EZ Disruption
109 / 217
C
EZ Disruption
128 / 217
2/9/2022, OD
IR&OCT 55° ART [HR] ART(17) Q: 33
D
EZ Disruption
109 / 217

Supplementary Fig. 7. Reproducible visual streak damage using the scleral patch (A-D) Left panels: lower magnification OCT evaluation at the terminal timepoint before enucleation show impact area (arrowhead) and ellipsoid zone (EZ) disruption (rectangles). Right panels: higher magnification OCT images confirm the absence of the ellipsoid zone (arrowhead).

				Damage extent CR area					Damage extent VS area			
Number of eyes (n)	Pressure used (PSI)	Calculated Velocity (Vm/s)	Impact location	Sclera	Choroid	RPE	Retina	Cornea	Sclera	Choroid	RPE	Retina
3	20	40.5	Cornea	-	-	++	+++	+++	-	-	+	+++
3	20	40.5	Limbus	-	-	-	-	+++	-	-	-	Dialysis
3	15	33	Sclera	-	-	-	+	-	-	-	-	+
3	17	37.5	Sclera	-	-	-	++	-	-	-	-	+
3	19	39.5	Sclear	-	+	+	++	-	-	-	-	++
3	25	48	Sclear	-	+++	+++	+++	-	-	+	++	+++

Key: - No damage, + Minimal damage, ++ Medium damage, +++ Significant damage
